# Cannabis use disorder: an overview of treatment approaches in Europe

**DOI:** 10.1007/s00406-025-01964-7

**Published:** 2025-03-04

**Authors:** Eva Hoch, Monika Murawski, Marica Ferri, Daniel Feingold

**Affiliations:** 1 Department of Psychiatry and Psychotherapy, University Hospital of Munich, LMU Munich, 80336 Munich, Germany; 2 IFT Institut für Therapieforschung, Centre for Mental Health and Addiction Research, Munich, Germany; 3 Department of Psychology, Charlotte Fresenius University, Munich, Germany; 4 Public Health (EUDA), Lisbon, Portugal; 5 Psychology Department, Achva Academic College, Arugot, Israel

**Keywords:** cannabis use disorder, Treatment, Europe, Review

## Abstract

Between 8 and 22% of lifetime cannabis users develop Cannabis Use Disorder (CUD). It is the most frequent reason for first-time drug-related treatment admissions in Europe. Many countries have general substance use treatment programs for individuals with cannabis use disorders. This study presents an updated overview of cannabis-specific treatment availability across Europe. Data on treatment programs in 27 EU member states, United Kingdom, Norway and Turkey was gathered. The study used a mixed-methods approach, combining (1) a quantitative survey among the National Focal Points of the European Drugs Agency (EUDA), (2) a qualitative analysis of “Drug Workbooks 2021” and ‘Treatment Workbooks 2020 and 2021’ published by the National Focal Points of the EUDA. Data for 30 countries on the European continent was analyzed. Sixteen countries reported the existence of specific cannabis-specific programs. Fifteen countries provided specific face-to-face interventions, which mostly have limited national coverage. Cannabis-specific online-treatment has been used more systematically since the COVID-pandemic in some countries. Automated and brief web-based interventions have emerged with a large potential to cover the needs of many clients in rural areas. First Cannabis Clinics opened, but specific forms of treatment for vulnerable target groups (e.g. adolescents, people with mental disorders) are generally still rare. Most programs are not evidence-based. In sum, some growth in cannabis-specific treatments has been observed in the past decade in Europe. Their coverage is still limited.

## Introduction

Cannabis is the most commonly used psychoactive substance in the world following caffeine, nicotine and alcohol [26]. In 2023, the European Monitoring Centre for Drugs and Drug Addiction (EMCDDA) estimated that in the European Union (EU) 22.6 million adults aged 15–64 (8% of this age group) had used cannabis in the past year, with 15.3 million of those users aged 15–34 years (15.1%). Cannabis use in this age group varied widely across Europe, ranging from 23% in Czechia to less than 2% in Turkey [11]. The 2019 ESPAD study showed that 16% of 16-year-old students had used cannabis at least once, with boys reporting more lifetime use than girls (18% vs. 13%). The highest rates of use were in Czechia (28%), Italy (27%), and Latvia (26%), while the lowest rates were in Kosovo, North Macedonia, Iceland, and Serbia (3%–7%) [20].

Cannabis remains illegal for recreational use in most European countries. Some countries have legalised adult cannabis under strict regulations, such as Malta, Luxemburg and Germany. Other countries,  such as the Netherlands and Spain, have decriminalized or tolerated limited use.  Medical cannabis is legal in several European countries, including Germany, Italy, UK and Denmark [6].

Between 8 and 22% of lifetime cannabis users will develop Cannabis Use Disorder (CUD) [8, 15, 17]. The risk of cannabis dependence rises to 33% (22–44%) among young people who use cannabis regularly (i.e. weekly or daily) [16]. Higher risk of CUD is also reported for men, individuals with comorbid psychiatric disorders and those suffering from adverse childhood events [4]. In 2022, 92,000 of all clients entering specialist drug treatment did so due to problems related to cannabis use (36% of all treatment entries), with about 58,000 entering for the first time [7]. Further, Cannabis was the most frequently cited main problem drug by new treatment clients in Europe (43% of all first-time treatment entrants) [7]. This indicates high rates of CUD, which are also observed in other parts of the world [12]. Cognitive behavioural therapy (CBT), motivational enhancement therapy (MET) and contingency management (CM) can substantially reduce cannabis use and cannabis-related problems [3], but enduring abstinence is not a common outcome. No pharmacotherapies have been approved for cannabis use or CUD [21].

A 2015 EMCDDA-report mapped the availability of cannabis-specific treatment in the EU, indicating that there are multiple addiction and mental health service responses to CUD [5]. These included self-help, motivational enhancement, brief interventions and counselling, cognitive-behavioral therapy and family therapy, which are often delivered in the community, but also to in-patients [5]. Moreover, a growing number of digital interventions target problematic cannabis use and predictors of effectiveness were discussed. This report aims to provide an updated overview of cannabis-specific interventions across Europe. We then provide an in depth analysis in each European country. The focus of this overview is on theory-driven and structured psychosocial treatments which are tailored to the specific problems and needs of individuals with cannabis-related problems. Such interventions have shown higher effectiveness compared to unstructured interventions or no treatment at all in individuals with CUD [9].

## Methods

### Procedure

Data on cannabis-specific treatment programs in 27 EU member states, United Kingdom, Norway and Turkey, were retrieved from several EMCDDA sources. From 2022 to 2023, data search, identification, and analysis were conducted by two researchers in Germany and one researcher in Israel on behalf of the EMCDDA, since 01. July 2024 known as the European Drugs Agency (EUDA). Treatment programs were included in our review if they were: (a) structured, i.e., included some form of instructions, manual or guidelines; (b) theory-driven, i.e., based on at least one theoretical mechanism of change (e.g., behavioural, emotional) which was previously conceptualized; and (c) specific, i.e., provided exclusively to individuals with cannabis-related problems and tailored to their specific needs. Due to these inclusion criteria, preventive interventions, and harm-reduction public-health strategies are not within the scope of this work and were therefore not included in our review.

1. In a first step, we searched all “Drug Workbooks 2021” and ‘Treatment Workbooks 2020 and 2021’ for information on cannabis use and psychosocial treatment for cannabis use disorders. 'Drug Workbooks' and 'Treatment Workbooks' are annual reports prepared by the National Focal Points of the EUDA information network on drugs and drug addiction. The National Focal Points were created at the same time as the EMCDDA. Members of the network are designated national institutions or agencies responsible for data collection and reporting on drugs and drug addiction from all EU Member States, Norway, Turkey, and the European Commission (https://www.euda.europa.eu). From 1 January 2021, the UK ceased to be a member of the network of National Focal Points. In order to obtain data for this study, experts in the field were contacted directly.
  The drug and treatment workbooks are internal EUDA documents used to retrieve qualitative and quantitative information from the reporting countries, as they often include detailed description and online links to specific treatment programs available in each country.
2. As the workbooks do not systematically report on cannabis-specific treatment programs available, we conducted a “Survey on Cannabis-Specific Treatments (SCST)” among the members of the European National Focal Points, Norway, and Turkey in a second step, to further explore the availability and nature of such programs. We developed a brief 5-item- questionnaire to collect more detailed information on the type of counselling and treatment approaches available in each of the EU countries. The questions were: “1. What type of cannabis-specific treatment is offered, if any?; 2. What type of provision is available for such programs (face-to-face/online)”; 3. What is the coverage of the program (full: nearly all people in need of help would obtain it; extensive: a majority but not nearly all of them would obtain it; limited: more than a few but not a majority of them would obtain it; rare: just a few of them would obtain it)”, 4. “Is there a treatment protocol /manual for the program? If so, please provide it in a separate file/ attachment”; and 5. “Is an implementation of cannabis-specific interventions planned in the next three years (yes/no)”. Additional information which was provided by respondents in their reply to question 4 was screened by the researchers in order to extract further details on the nature of the mentioned treatment programs, focusing on structure, specificity and underlying theory of available treatment programs.
3. In a third step, we further approached members of the National Focal Point in cases where data was missing on the structure, underlying theory/concept or specificity of cannabis-related treatment programs. In this step, members of the National Focal Point were contacted by E-mail in order to provide additional information or share material on national programs for cannabis use disorders.

### Data synthesis

Upon completion of these three steps, data from each country was compiled and reviewed by the authors in order to determine whether the mentioned programs met inclusion criteria. Controversial cases were discussed until consensus was achieved.

## Results

### The European picture

Reports on the drug situation in the EU member states indicate that health care systems have responded to cannabis-related treatment demand (Table 1). Based on our research, two treatment approaches for CUD are evident throughout Europe. (1) General substance use treatment, which is tailored to the individual needs of the treatment seeking cannabis user. (2) Cannabis-specific interventions in addition to general substance use treatment. This approach targets treatment to the specific characteristics of a person with CUD, as well as to specific cannabis-related social or health risks. Therapeutic strategy and materials also match the characteristics of the cannabis users (e.g., age, gender, peer group, culture).

**Table 1:** Availability of cannabis-specific treatment in the European Union, the UK, Norway and Turkey, by country

	Cannabis-specific treatment available	CST coverage*	Implementation of CST planned?	Type of treatment offered	Source of information
Austria	Yes	Face-to-face: limited Online: unknown	n.a	CANDIS www.candis-projekt.de Online: CANreduce www.canreduce.at	Workbooks SCST Personal communication
Belgium	Yes	Face-to-face: limited Online: full	n.a	Inpatient treatment: “Cannabis Clinic” (e.g. Multidimensional Family Therapy) Online: www.cannabishulp.be www.druglijn.be www.drughulp.be	Workbooks SCST
Bulgaria	No	n.a	No information	n.a	Workbooks SCST
Croatia	No	n.a	Yes	n.a	Workbooks SCST
Cyprus	No	n.a	No	n.a	Workbooks SCST
Czech Republic	Yes	Online: full	n.a	Online: www.koncimshulenim.cz/ (“I’m Quitting Pot”)	Workbooks SCST
Denmark	No	n.a	No information	n.a	Workbooks
Estonia	Yes	Face-to-face: limited to the capital Tallinn Online: full	YES	Outpatient and inpatient treatment; Online: VALIK (Choice) based on “Screening, Brief Intervention and Referral to Treatment (SBIRT)”	Workbooks SCST Personal communication
Finland	No	n.a	No information	n.a	Workbooks SCST Personal communication
France	Yes	Face-to-face: limited to the Département Saone-et-Loire	n.a	CANDIS	Workbooks SCST Personal communication
Germany	Yes	Face-to-face: limited Online: full	n.a	Realize-It, CANstop, CANDIS; Online: Quit the Shit	Workbooks SCST
Greece	No	n.a	No information	n.a	SCST
Hungary	No	n.a	No information	n.a	Workbooks SCST
Ireland	No	n.a	No information	n.a	Workbooks SCST
Italy	Yes	Face-to-face: limited to the region of Trentino-Alto Adige	n.a	CANDIS	Workbooks Personal communication
Latvia	No	n.a	No information	n.a	Workbooks SCST
Lithuania	No	n.a	No information	n.a	Workbooks SCST Personal communication
Luxemburg	Yes	Face-to-face: limited	No information	CHOICE CHOICE18 +	Workbooks SCST Personal communication
Malta	No	n.a	No information	n.a	Workbooks SCST
Netherlands	Yes	Face-to-face: unknown Online: currently as part of a study	n.a	Online: ICan	Workbooks SCST
Norway	Yes	Face-to-face: limited	n.a	Cannabis detox program (CDP) (formerly known as "Out of the fog")	Workbooks SCST
Poland	Yes	Face-to-face: extensive	n.a	CANDIS	Workbooks SCST
Portugal	Yes	Face-to-face: limited to a clinic in Porto	n.a	CANDIS	Workbooks SCST Personal communication
Romania	Yes	Face-to-face: limited	n.a	Inpatient treatment: Color Mind Clinic https://color-mind.ro/excelenta-in-adictii/tratament-dependente/dependenta-de-cannabis/	Workbooks SCST
Slovakia	No	n.a	No information	n.a	Workbooks SCST
Slovenia	No	n.a	No	n.a	Workbooks SCST
Spain	Yes	Face-to-face: limited to one clinic in Barcelona Online: currently as part of a study	n.a	Outpatient treatment: CANDIS Online: CANreduce-SP	Workbooks SCST
Sweden	Yes	Face-to-face: unknown Online Beroendecentrum Stockholm: full Online D17: limited	n.a	Online: Stockholm Centre for Dependency Disorders (Beroendecentrum Stockholm) Online: D17	Workbooks
Turkey	No	n.a	No	n.a	Workbooks SCST
United Kingdom	Yes	Face-to-face: limited to patients under the care of the South London and Maudsley NHS foundation Trust, London (SLAM) Online: potentially full	n.a	Treatment in the community and hospital: Cannabis Clinic for patients with psychosis and CUD, including special online program “CCP PEER group”	Personal communication

*CST* cannabis-specific Treatment, *SCST* Survey on Cannabis Specific-Treatments, *n.a.* not applicable

*Rating by National Focal Points. Rating scale: “full”: nearly all people in need of help would obtain it; “extensive” a majority but not nearly all of them would obtain it; “limited”: more than a few but not a majority of them would obtain it; “rare”: just a few of them would obtain it

**Fig. 1:**
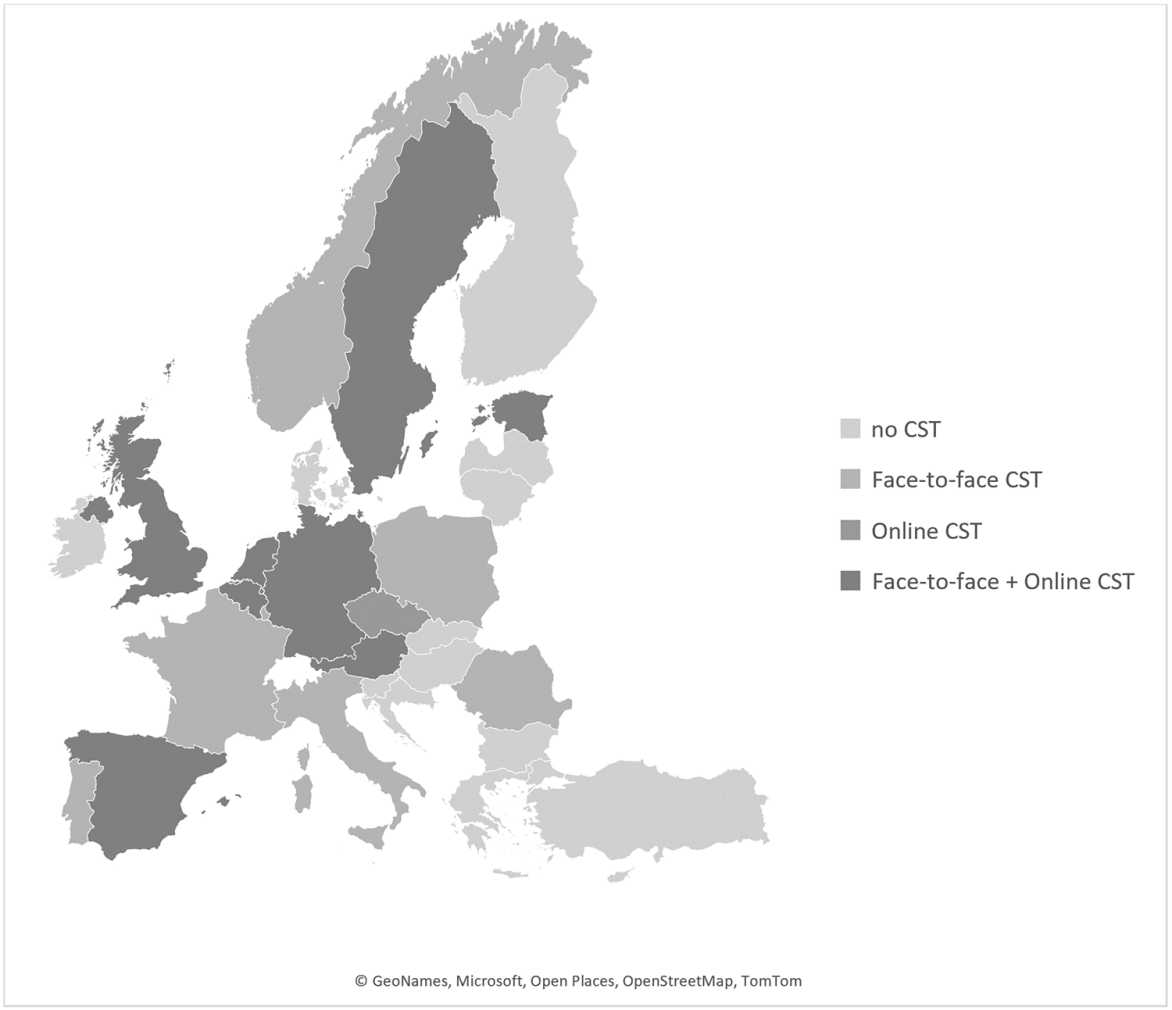
Availability of Cannabis-specific treatment in the European Union, the UK, Norway and Turkey, by type of treatment

The United Kingdom and all 29 countries which are affiliated with the EUDA evote considerable resources to treating CUD through general substance use programs. These are offered to patients voluntarily, or as an alternative to punishment for those who are undergoing legal procedures related to cannabis possession. Of all 30 countries analyzed, 16 provided information on kinds of cannabis-specific programs. Fifteen countries provide interventions that are delivered face-to-face; and 9 countries established online information, self-help or therapist-guided programs (Fig. 1).

The national coverage for in-person treatments has been described as “rare” or “limited” in some countries (e.g., Austria, Estonia, France, Italy, Spain, UK). In these countries, cannabis-specific treatment programs are available in specific regions or cities. For example, Screening, Brief Intervention and Referral to treatment (SBIRT) is primarily delivered in the Estonian capital Tallinn. In other countries (e.g., Belgium, Luxemburg, Norway, Romania Germany and Poland), several cannabis-specific approaches are available, free of cost, but experts described the coverage among all individuals seeking care as “limited”. During the Covid-19 pandemic many treatment centers provided individual or group sessions online.

Recently, web-based treatment programs have become more available in Austria, Belgium, Czech Republic, Sweden, Spain, UK, Estonia, Germany, and the Netherlands. These programs (e.g. VALIK, CANreduce, SBIRT, Quit the shit) provide information on cannabis (and other drugs), brief assessments, self-help and in some cases meetings with a drug counselor or therapist. These approaches are free of cost and benefit are are highly available. Even when administered at a local level, they can be used by people in other regions of the country.

First designated cannabis clinics had emerged in Belgium, Romania and in the UK. These centers have a specific focus on cannabis use disorders and psychoses/schizophrenia or other comorbid mental disorders. They provide clinical assessment, detoxification, inpatient treatment, day care and longer-termed rehabilitation. Intervention is tailored to the patient's individual needs and an interdisciplinary therapeutic team addresses the full spectrum of medical, social, and psychological needs in order to foster positive long-term treatment outcomes (e.g., abstinence from cannabis, reduction in cannabis use, positive prognosis of the psychosis, participation and satisfaction with life).

Evidence-based cannabis-specific interventions were in the minority in the “Workbooks” or in the Survey on Cannabis-specific Treatments (SCST) which was conducted among the National Focal Points. Several approaches are currently being evaluated in scientific studies, for example the online program “ICAN” in the Netherlands [22]. Other programs have scientifically proven benefits (e.g., “I am quitting pot” in Czech Republic, koncimshulenim.cz), or are being translated and adapted to other countries/languages, for example the Swiss online-program CANreduce, or the CANDIS program from Germany [13, 14].

Little information was available on cannabis-specific interventions for vulnerable groups such as children and young adolescents. Multi-dimensional Family Therapy (MDFT) was successfully tested and implemented in various EU countries about 15 years ago (e.g., in the Netherlands, Belgium, Germany, France), but not mentioned in the 2022 reports. In Sweden, two online-based programs exist—one for individuals who are 15–17 years old that involves parents so that the individual can receive support from a parent or guardian, and one for individuals who are 18–25 years old. Moreover, few countries provided information on treatment of CUD in prisons, either general (e.g., in Bulgaria) or cannabis-specific (e.g., Germany). In order to get a clearer picture of the treatment landscape for those with a cannabis use disorder and associates mental, physical and social problems, country profiles are provided in the next section.

### Country profiles

#### Austria

The Institute for Drug Prevention of the Vienna Addiction and Drug Prevention launched the digital mental health platform “mindbase” (https://mindbase.at/) in December 2019 and intensively advertised it in the pandemic year 2020. The platform offers information on mental health and addiction. A series of online self-help tools is provided online. They can be used free of charge and anonymously. One of these online self-help programs is CANreduce, (https://www.canreduce.at). It was developed and tested in Switzerland [2] with the aims of reducing or stopping cannabis use.

The CANDIS-program (https://www.candis-projekt.de) is run by the outpatient drug counselling and treatment center Z6 in Innsbruck (https://www.drogenarbeitz6.at). In Cathartunia, the health care provider “pro menthe” organized two 2-day-trainings on the CANDIS-program for its member services in 2018 and 2022. About 45 drug addiction experts took part at the workshops. The program is implemented in outpatient and inpatient drug treatment services and in day care and prisons.

#### Belgium

Since 2006 cannabis-specific treatment has been provided through one specific unit treating CUD in Brussels at the Brugmann hospital, called “cannabis clinic” which has a section for adults and a section for adolescents. They offer individual and group therapy, cognitive behavioural therapy (CBT) and multidimensional family therapy (MDFT) [24] especially for adolescents.

In Belgium, e-Health services are also provided. A cannabis-specific website is available in Dutch at www.cannabishulp.be. The Flemish helpline (“De DrugLijn”, www.druglijn.be) and Infor-drogues provide support by telephone and Email. They also offer online assessments and online self-help programmes for cannabis users. They also have a service to give support and guidance: infor-drogue provides this service face-to-face and De Druglijn has an automated online self-help program called “Dash”. The national coverage of the affected population is rated as comprehensive.

#### Bulgaria

An expansion of the treatment system for substance use disorders has produced new inpatient centres and units in prisons to provide treatment for people with substance use disorders. Psychotherapy and psychosocial rehabilitation are delivered face-to-face. Counselling is provided face-to-face and online. Coverage is considered as “limited”. Currently, cannabis-specific interventions are not available and the National Focal Point does not know if any planned cannabis-specific treatment being implemented within the next three years.

#### Croatia

In Croatia, people with CUD are primarily treated in the health care system. In accordance with national policy, the treatment of cannabis users aims at early identification, timely provision of treatment to as many abusers as possible, professional medical supervision, easy access, and individual approach suiting the characteristics of a patient, including “low intensity” programs for persons who are interested in treatment.

Services for mental health protection, addiction prevention and outpatient treatment and hospitals are also available. They offer various interventions, such as supportive psychotherapy, psychoeducation, behavioral psychotherapy, family psychotherapy. Psychosocial treatment is conducted in the social welfare system, therapeutic communities, and associations. To date, no cannabis-specific interventions have been offered. According to the National Focal Point Croatia is in the process of adopting a new National Strategy in the Field of Addiction 2022–2030 after which an Action Plan in the Field of Addiction will be launched for the period 2022–2026. It foresees the development of a wide range of specific treatment interventions, including those for cannabis. So, if all goes as planned, Croatia may be able to implement cannabis-specific treatment within the next 3 years.

#### Cyprus

In Cyprus, cannabis treatment was improved by the consolidation and expansion of the cooperation protocol between the police and treatment centers, and the noteworthy efforts of the Drug Law Enforcement Unit, to refer first time drug offenders to treatment. More available treatment increased the number of clients. There is no cannabis specific treatment currently offered in Cyprus and it has not been included in the Action Plan 2021–2024 accompanying the National Strategy 2021–2028.

#### Czech Republic

Since 2013, SANANIM civic association operates the website www.koncimshulenim.cz (“I’m Quitting Pot”) that is targeted specifically to cannabis users. It offers information on cannabis and the risks associated with its use. Advice is given on how to reduce consumption or quit. The site also offers a self-assessment test to detect problem cannabis use and an online treatment program for 8 weeks, the first of its kind in the Czech Republic. Coverage of the online program is considered as “full” by the National Focal Point. The field of cannabis and cannabinoids is one of the special topics of the new National Strategy 2019–2027 approved in 2019.

#### Denmark

People with CUD as other substance use disorders are treated in specialized outpatient treatment programs in Denmark. If a person does not want to avail him or herself of the public treatment programs, they can pay for the treatment by a private psychologist. No information is available on cannabis-specific interventions.

#### Estonia

The “VALIK” (“Choice”) program for adults with CUD has been available in Estonia since 2018. This outpatient treatment program is based on the module “Screening, Brief Intervention and Referral to Treatment (SBIRT)”. It is offered in face-to-face format in the country's capital area of Tallinn. Since 2020 a web-based version has become available to all Estonian residents. The program is offered to patients voluntarily, or as an alternative to punishment for those charged with cannabis possession. Currently the program is being adapted to the adolescent population. Estonia also offers designated inpatient drug treatment for patients with CUD with an average duration of four weeks. Further implementation of a brief cannabis-specific treatment for minors aged < 17 is expected soon.

#### Finland

To the best of our knowledge, no cannabis-specific treatment is offered in Finland. Individuals with cannabis-related problems are treated in outpatient and inpatient drug treatment centres, which address all types of substance-related problems.

#### France

In France, cannabis users comprise the majority in referral to treatment in outpatient youth addiction clinics (CJC) and inpatient drug treatment centers (CSAPA). No cannabis-specific treatment is available in these units. Since January 2021, implementation of the CANDIS program has been implemented in 6 outpatient addiction clinics in the Département Saône-et-Loire. A French version of the therapist-manual is available, but it has not been published so far. A process evaluation and assessment of its transferability is being undertaken. Recently, the Ministry of Health launched a working group focusing on implementation of cannabis-specific treatment in France. In the early 2000ies, France also participated in the multinational project INCANT (INternational CAnnabis Need for Treatment), testing the effectiveness of Multidimensional Family Therapy (MDFT) in a controlled trial [24]. No information could be gathered on the recent coverage of MDFT in France. 

#### Germany

In Germany, people with CUD are mostly treated within general outpatient substance use services. Cannabis-specific approaches and specialized treatment settings are also available. The decision as to the scope of treatment, setting and the appropriate therapeutic interventions depends on age, severity of dependence and acute comorbid disorders. Treatment can take place in the context of youth welfare, outpatient or inpatient addiction treatment centres, psychotherapy practice, or in psychiatric hospitals.

Cannabis-specific concepts are available as early and brief counselling concepts (e.g., Realize-It; https://realize-it.org), treatment for adolescents and adults with or without comorbidity (e.g., CANDIS) [13, 14], intensive interventions for youth with cannabis use and multiple problems and their families (e.g., Multi-dimensional Family Therapy, MDFT) [24] or group sessions in prison (e.g., CAN Stop) [1]. All of these approaches are partially available. They were developed and evaluated in clinical trials and have been implemented in transfer studies in Germany since 2006. A web-based program is available, too (e.g. Quit the shit; https://www.quit-the-shit.net).

People with CUD, severe dependence, heavy symptoms of withdrawals, multiple drug use or comorbid mental disorders are treated in specialist detoxification units in psychiatric hospitals. They may receive longer addiction rehabilitation in outpatient or inpatient services using integrative therapy concepts if needed.

#### Greece

There is currently no cannabis-specific treatment in Greece. Individuals with cannabis-related problems are referred to general substance use treatment programs that provide psychosocial care.

#### Hungary

Individuals who are referred to treatment for cannabis-related problems are admitted to an inpatient or outpatient general drug treatment service throughout the country. The Hungarian Ministry of Health has published national guidelines for clinicians who work with patients reporting CUD. In several treatment services, some cannabis-specific elements are integrated into treatment, yet no cannabis-specific treatment is available in the country.

#### Ireland

The vast majority of individuals with cannabis-related problems are admitted to general drug treatment services. To the best of our knowledge, no cannabis-specific treatment is available in Ireland.

#### Italy

The vast majority of patient who are admitted to treatment for a CUD are treated in general inpatient or outpatient treatment units for substance use disorders. In 2020, the treatment provider “La Strada/Der Weg” (located in Bolzano) started to implement a cannabis-specific intervention in Trentino-Alto Adige (https://www.lastrada-derweg.org). In this region in northern Italy, several units offer the CANDIS program in Italian or German language to patients whose primary drug of abuse is cannabis.

#### Latvia

In Latvia, individuals with CUD can get help in outpatient or inpatient general addiction treatment services. There is currently no specific treatment approach for CUD in Latvia. No information is available on whether cannabis-specific approaches are planned.

#### Lithuania

Lithuania offers treatment programmes that involve counselling, detoxification, psychosocial interventions and rehabilitation for individuals with CUD. The majority of those in need of treatment for cannabis use problems have access to treatment. No additional information is available on the specific types of treatment programmes and their setting.

#### Luxemburg

Cannabis-specific treatment is available in Luxemburg, predominantly in outpatient settings. The non-government treatment agency IMPULS (https://www.solina.lu/facilities/impuls/) offers two outpatient treatment programs, free of charge. They target cannabis users who are referred to treatment as an alternative to criminal record registration. These programs are titled CHOICE (for patients aged 12–17) and CHOICE18 + (for patients aged 18–21). They comprise of four group sessions and aim to provide a more critical view towards cannabis use and prevent the further development of drug abuse and addiction. A long-term evaluation of CHOICE was conducted over 4 years [25]. Considering an expected national policy change in cannabis regulation, the “CHOICE” programmes will be revised and further adapted to patients' needs. Additional national treatment programs for adolescents with cannabis-related problems are likely to be developed over the next 3 years in collaboration with the national centre for addiction prevention, the Ministry of Education and Youth, and the Ministry of Health.

#### Malta

No cannabis-specific treatment is available in Malta. Patients with CUD receive general substance use treatment in inpatient and outpatient treatment units.

#### Netherlands

A ‘Multidisciplinary guideline to treat disorders are related to the use of cannabis, cocaine, amphetamines, ecstasy, GHB and benzodiazepines’ was developed in the Netherlands. The guideline recommends cognitive behavioural therapy in combination with motivational interviewing, or contingency management. Young problem-users of cannabis may also receive multidimensional family therapy. The providers for addiction care in the Netherlands offer a variety of these treatment programs for individuals with CUD. Because there are generally no complications associated with cannabis detoxification, inpatient detoxification is usually not required.

The Trimbos Institute recently developed an online cannabis reduction intervention (ICan, see https://www.trimbos.nl/aanbod/interventies/) based on the Screening, Brief Intervention and Referral to treatment (SBIRT) approach. The mobile web app is based on motivational interviewing and cognitive behavioral therapy. It focuses on increasing treatment adherence with guidance via email, push notifications and WhatsApp. Up to this point, the ICan intervention has only been available through participation in the randomized controlled trial [22]. In the early 2000s, the Netherlands participated—together with Belgium, France, Germany, and Switzerland in a transnational clinical trial to successfully test the Multidimensional Family Therapy (MDFT) in controlled trial named INCANT (INternational CAnnabis Need for Treatment) [23]. The intervention has since been implemented. No information was available on the national coverage of MDFT in the Netherlands.

#### Norway

Norway offers online tests and self-help programs (the latter developed in the Netherlands and translated into Norwegian) whereby individuals can anonymously receive help with managing mild to moderate drug problems. The pages at www.rushjelp.no contain self-tests for cannabis, alcohol and cocaine use. The tests are conducted free of charge and anonymously to lower the threshold for drug-treatment. According to the score a drug user receives, he or she is invited to take part in a self-help program on the internet. It is “user-controlled”, i.e. participants determine the length of the program (4–6 weeks) and treatment goals (e.g., abstinence or reduction). As part of the self-help program, it is also possible to communicate with other users, to share experiences and motivate each other. For those who want further assistance during or after the course, the email address and telephone number of The Bergen Clinic Foundation is always available within the program (https://bergenregional.com).

A cannabis-specific program previously called “out of the fog” is now called “Cannabis Detox Program (CDP)”. Several municipalities have implemented the program and a free mobile app is also developed to aid the program (https://apps.apple.com/no/app/hap/id1145093225).

#### Poland

In the past decade, the National Bureau for Drug Prevention has supported the implementation of the CANDIS treatment program in Poland (www.candisprogram.pl). Addiction treatment experts from drug treatment facilities from all over the country have been trained on the program. All clinicians received additional training in motivational enhancement therapy as an adjunct to the CANDIS Program. The program is usually applied face-to-face. In case of an individual request by the participant online sessions are possible. However, not all facilities provide the program.

#### Portugal

Although treatment of opioid use remains a focus of the drug treatment system, special programs for cannabis users have also been created. CANDIS was implemented at a university hospital in Porto, and there is a Portuguese translation of the CANDIS treatment manual.

#### Romania

In Romania, treatment centers address all drug users. Cannabis treatment programs have become available nationwide, both in the community and in prisons. They have been implemented by the National Antidrug Agency, by the Color Mind Clinic and by various NGO`s. Some of these programs are free of charge, some have to be paid for. First, all drug using individuals are clinically assessed medically, psychologically and socially. Depending on the result of the evaluation, they receive specific treatment programs, e.g., for cannabis. At the Color Mind Clinic (https://color-mind.ro/excelenta-in-adictii/tratament-dependente/dependenta-de-cannabis/), for example, a therapeutic team of a case manager, an individual therapist and a psychiatrist work together to address the full spectrum of effects and consequences of CUD in the life of an individual. The case manager looks at the family, social, professional and legal aspects and their role in recovery (facilitating factors and obstacles to recovery). The psychiatrist checks physical and mental health, evaluates the risk of withdrawal and assesses whether and how detoxification should be carried out. The individual therapist helps to identify the client's personal resources, level of awareness of the condition, motivation to change, confidence in their own potential for change and the importance they place on recover.

There are no cannabis-specific treatment centers for CUD in Romania.

#### Slovakia

Children and adolescents receive primary prevention from specialised addiction services. Those who are experimenting with drugs. can recieve counselling from these services. An important role in Slovakia plays is layed by school counsellors and psychologists, who operate in all elementary and secondary schools. The programs for young people with drug problems focus to new trends in non-opioid drug use and poly-drug use. Currently, there is no information on whether cannabis-specific programs are available in Slovakia.

#### Slovenia

Cannabis users can enter the DrogArt counselling program (https://www.drogart.org/). They can also seek help in all general drug treatment programs that provide counseling, brief interventions and treatment, harm reduction programs or social rehabilitation programs. Slovenia is developing programs for individual target groups, but these are implemented within existing drug addiction treatment programs. This is considered the most suitable solution for a small country because of the difficulties in developing separate treatment networks for specific substance use disorders. Access to existing programs is good, as none has a waiting list. However, there are some problems in regions where there are no such programs and patients need to travel to distant facilities.

#### Spain

Spain is a decentralised country and treatment of substance use disorders within the health care system is carried out by the “Comunidades Autonomas” (regions). Any treatment follows scientific evidence and depends on patient needs. Some protocols have been developed and used as routine practice (e.g., https://socidrogalcohol.org/proyecto/guia-clinica-de-cannabis/ or https://www.redalyc.org/articulo.oa?id=83949782002).

Treatment programs are carried out taking into account the specific characteristics of each patient and not the substance itself. The interventions are biopsychosocial and involve multidisciplinary teams. In the case of minors, interventions (individual or group) aim to prevent chronic or higher risk use. These interventions are carried out in accordance with specific protocols and take place at different times and/or places. Treatment with minors is as individualized as possible and takes into account the personal, family and social characteristics surrounding the minor. Currently, a study is testing the effectiveness of the Spanish version of CANreduce 2.0 (CANreduce-SP) in reducing both the frequency and quantity of cannabis use in problematic users and in assessing whether adding psychological support increases its effectiveness [19]. In the university hospital, the CANDIS manual [13, 14] was translated to Spanish and the program implemented in an outpatient treatment service for individuals with CUD in the university hospital in Barcelona (https://www.centrobonanova.com/tratamientos/cannabis).

#### Sweden

Cannabis treatment is available online for the whole country through the Stockholm Centre for Dependency Disorders (Beroendecentrum Stockholm). The program is based on CBT and MI. For young people, Maria Ungdom has two online-based programs. One is for individuals who are 15–17 years old. It  also involves parents so that adolescents can receive support from a parent or guardian. The second program is for young adults aged 18–25 years.

In the national Swedish guidelines for problem drug use and dependence (published in 2019 by the National Board of Health and Welfare), there is a recommendation for a digital intervention for cannabis use and dependence, called D17. This treatment is not prioritized in Swedish health care, because of the lack of evidence on its effectiveness. According to the guidelines, it should be used in exceptional cases.

#### Turkey

In Turkey, there is no specific cannabis treatment model. Outpatient and inpatient services are available for individuals with substance use disorders. A “Training Program on Fight Against Addiction” was prepared in 2015 and updated in 2018 to increase the effectiveness of the services and family physicians. The program also included a train-the-trainer program. No information is currently available on plans to develop cannabis-specific interventions.

#### UK

In the United Kingdom, several cannabis-specific approaches have become available in the past decade. “Marijuana Anonymous” is a self-help group which is based on the 12-step model. It is potentially available online and face-to-face nationally.

In London, a Cannabis Clinic for patients with psychosis (CCP) was founded (https://maudsleylearning.com/courses/cannabis-clinic-for-patients-with-psychosis-live-groups-virtual-learning-environment/). It is limited to treating dual diagnosis patients with psychosis and CUD under the care of the South London and Maudsley NHS foundation Trust, London (SLAM). The clinic provides a special online program “CCP PEER group” with psychoeducation and peer support once a week: International speakers deliver a 10-min talk on a relevant topic. The Cannabis Clinic for patients with psychosis hopes to expand to other NHS trusts in the UK.

## Discussion

In the past decade, the need of treatment for CUD and its related mental, physical or social problems has increased [10]. This mapping study was conducted on behalf of the European Drugs Agency.

It reveals, that on the European continent, there has been some growth of treatment provision since a similar analysis was carried out 10 years ago [5]. Addiction treatment for CUD is more available now. New and more treatment services have been established in many EU countries. In all of them, patients with CUD are integrated into the existing treatment system for substance use disorders. In half of the European states, specific services for cannabis users are provided, mostly via face-to-face programs at regional or local level. Online-treatment has been used more systematially since the beginning of the COVID-pandemic [18]. Automated and brief web-based interventions have emerged with a large potential to cover the needs of many clients (e.g., in rural areas). It is important to mention, that 50% of countries with CST (n = 9) had also at least on online program (5 to 6 with full national coverage).

Nevertheless, there is still considerable room for improvement. There still is a large treatment gap for severely ill patients (e.g., with cannabis use and psychoses or other severe mental illness), and those with specific treatment needs (e.g., children and young adolescents, women, pregnant women, elderly people). Programs also need to engage more with families, partners and the social environment. They should be provided in specific settings where CUD is common (e.g., criminal justice system, prisons, schools, job centers, hospitals, emergency departments). A desirable future direction is the implementation of more evidence-based interventions that have shown benefits on abstinence, reduction of cannabis use, well-being and other clinical outcomes.

The picture on treatment for CUD in Europe remains incomplete. This report was built on the basis of a “Survey on Cannabis-specific Treatments (SCST)” conducted among the members of the European National Focal Points, Norway and Turkey.  Of 30 countries, 28 participated at the survey and completed the questionaire (completion rate: 96.7%).  National drug reports (published in the years 2021 and 2022) were systematically searched, too. These documents are annually submitted to the EUDA to describe the actual drug situation, recent data and trends in the respective EU countries. It is possible that more established cannabis-specific approaches exist at national, regional or local level, but have not been repeatedly be described in the recent reports.

Through personal contact and communication, more information was added to the reports based on the best knowledge of experts. Further, for the UK only personal communication with experts was available, which potentially limits the validity of the information.

A final limitation has to do with the fact that only few of the reported cannabis-specific interventions have been developed or tested in clinical studies. Little information is available on clinical outcomes or treatment manuals to provide insight into therapeutic strategies or treatment regimes. It may also be possible, that new studies are underway, that have not been reported or published so far.
